# Outcomes of clinico-radiologically advanced cancer (cT4b) of buccal mucosa: a retrospective analysis of 104 patients

**DOI:** 10.3332/ecancer.2022.1400

**Published:** 2022-05-26

**Authors:** Suvraraj Das, Rajeev Sharan, Anoop Attakkil, Saugata Sen, Kapila Manikantan, Prateek V Jain, Pattatheyil Arun

**Affiliations:** 1 Department of Surgical Oncology, Tata Medical Center, Kolkata 700160, India; 2 Department of Head and Neck Surgery, Tata Medical Center, Kolkata 700160, India; 3 Department of Radiology, Tata Medical Center, Kolkata 700160, India

**Keywords:** squamous cell carcinoma of head and neck, locoregional recurrence free survival, disease free survival, overall survival, CECT scan, cT4b lesions

## Abstract

Although guidelines recommend non-surgical management for cT4b patients, recent studies have shown that upfront surgery in carefully selected patients can be performed with acceptable long-term survival benefit. In this study, we analysed the survival outcome of curative intent treatment on cT4b patients. Data from 104 patients who were staged cT4b and underwent upfront surgery for squamous cell carcinoma of buccal mucosa were retrospectively analysed. Outcome measures were locoregional recurrence-free survival (LRFS), disease-free survival (DFS) and overall survival (OS). The study cohort comprised 104 patients who had a median age of 52.5 years (range 27–81 years) and included 81 males (77.9%). Thirty-six patients had masticator space involvement on final histopathology, designating them as pT4b. Contrast enhanced computed tomography scan demonstrated 91.67% sensitivity in identifying masticator space involvement, albeit with a lower accuracy of 31.7%. Pathologically, clear margins were achieved in 79 (76%) patients. 62 (59.7%) and 20 (19.2%) patients received adjuvant radiotherapy (RT) and adjuvant chemoradiotherapy respectively. 2-year LRFS, DFS and OS were 61.8%, 60% and 68.1%, respectively. On multivariate analyses, involved margins (hazard ratio (HR) 28.766, *p* = 0.006), pN2b status (HR 4.68, *p* = 0.027) and perineural invasion (PNI) (HR 3.001, *p* = 0.027) showed statistically significant impact on LRFS, involved margins (HR 28.859, *p* = 0.008) and pN2b status (HR 4.018, *p* = 0.004) affected DFS. Involved margins (HR 14.139, *p* = 0.023) and pN2b status (HR 3.166, *p* = 0.025) showed statistically significant impact on OS. In conclusion, upfront surgery is a feasible option for patients with carcinoma of the buccal mucosa with the involvement of the masticator space. Survival outcomes are better in patients where resection is achieved with clear margins, and regional disease is limited to a single cervical lymph node.

## Introduction

Almost two-thirds of all cancers in the oral cavity are present in advanced stages in the Indian subcontinent [1]. Compared to western countries where the tongue is the commonest site or origin, the gingivo-buccal mucosa predominates as the most prevalent subsite in our patients where consumption of betel nut, tobacco and areca nut have been implicated as aetiological factors [2]. Moreover, the proximity of the buccal mucosa to the masticator space and pterygoid plates results in early invasion of these structures, escalating the stage to T4b [3].

Contrast enhanced computed tomographic (CECT) scan of the neck remains the standard pre-operative imaging modality for malignancy of the buccal mucosa. Although CECT scan with 1.25 mm sections has shown to be highly sensitive and specific (sensitivity 82.6%, specificity 86.9%) for identification of mandibular invasion, its accuracy in diagnosing masticator space invasion has not been established so far [4].

Current National Comprehensive Cancer Network guidelines recommend clinical trials or non-surgical management for T4b disease in view of very advanced stage of the disease, difficulty in achieving R0 resection and increased surgical morbidity [5]. However, after the results published by Liao *et al* [6] showing favourable outcomes in infra-notch cT4b patients, surgical resection for selected patients has been the norm. Trivedi [7] have also proposed radical compartmental en-bloc resection in T4b disease to attain clear margins. However, extension to the infratemporal fossa defies attempts to delineate margins with accuracy as the area is bounded by bone on three sides and does not yield itself to clinical examination on the table and the surgeon is obligated to depend entirely on the accuracy of imaging modalities to guide his surgery on.

In this study, we analysed the outcomes of curative intent treatment for cT4b patients in terms of locoregional recurrence-free survival (LRFS), disease-free survival (DFS) and overall survival (OS).

## Material and methods

This retrospective cohort study comprised patients who underwent surgery for oral squamous cell carcinoma (OSCC) from August 2011 to June 2017. Patients who underwent tumour resection as the primary method of treatment and had primary tumour staged T4b clinico-radiologically or on definitive histopathology were considered eligible for inclusion. Patients who had primary lesions staged other than T4b had history of receiving prior treatment or if their preoperative CECT scan was unavailable were excluded from the study. A total number of 725 patients of OSCC were operated on in this period, of which 104 eligible patients formed the cohort for this study. Approval from the Institutional Ethical and Review committee was obtained prior to commencing the study vide Ref No EC/WV/TMC/19/21.

Demographic and tumour characteristics, adjuvant treatment, and clinical outcomes were taken directly from patient charts. In all cases, preoperative CECT scan of the neck was evaluated by a senior radiologist and was divided into two groups, viz., with or without involvement of masticator space (involvement of at-least one of masseter, medial pterygoid, lateral pterygoid or temporalis muscle). The radiologist was blinded to the final histopathology report.

Other study variables, namely, age, gender, tumour grade (well differentiated, moderately differentiated and poorly differentiated) pathological T classification and N classification, extra nodal extension (ENE), lympho-vascular invasion (LVI) and perineural invasion (PNI), were derived from definitive pathology reports, Information on adjuvant treatment with radiation or chemotherapy (any dosage or type of radiotherapy (RT) or chemo-radiation (CTRT) was recorded as ‘yes’ or ‘no’). Staging was based on the the American Joint Committee on Cancer (edition 7) [8] histopathologic risk factors, namely, pathological T and N classification, tumour site, tumour grade, ENE, LVI, PNI and surgical margin were considered for administration of adjuvant treatment by a multidisciplinary team.

Clinical outcomes were retrieved from the patient charts. LRFS was defined as the time between the day of primary surgery for OSCC and the date of pathological confirmation of locoregional disease recurrence. LRFS for patients without documentation of locoregional recurrence was censored on the last day of their follow up. DFS was defined as the interval between day of primary surgery for OSCC and the day of clinico-radiological confirmation of disease recurrence (locoregional or distant metastasis). DFS for patients without documentation of disease recurrence was censored at the last day of follow up. Death was also considered as an event for calculation of DFS and LRFS. The time for OS was determined between the day of primary surgery for OSCC and the date of death. OS for patients without documentation of death was censored at the last date that the patient had been known to have been alive.

Data analysis was done using Stata version 14.2 (Stata Corp). Kaplan–Meier survival curves were used to calculate LRFS, DFS and OS. Comparisons survival between groups were performed using Log-rank Tests. Multivariate Cox proportional hazards models were used to assess the prognostic effects of demographic and tumour characteristics and adjuvant treatment on survival outcomes. Variables with univariate *p*-values < 0.05 were included in multivariate models. Variable selection using both forward selection (*p* ≤ 0.05 to enter) and backward elimination (*p* > 0.05 to remove) was performed to obtain final models. The level of statistical significance for all tests was < 0.05.

## Results

The study cohort comprised 104 patients of which 81 patients (77.9%) were male and 23 were female (22.1%). The age of the patients ranged between 27 and 81 years, with a median age of 52.5 years. Median follow up of all the patients who were alive at the time of analysis was 28 months (range 0.5–76.5 months)

All patients underwent a CECT scan, which revealed 101 (97.1%) patients to have masticator space involvement. All patients had infra-mandibular notch disease on pre-operative CECT scan. 33 patients had masticator space involvement on which was corroborated with HPR. Likewise, there were three cases where masticator space was free on CECT scan, but final histopathology reported involvement of the masticator space. CECT scan identified the masticator pace involvement with 91.67% sensitivity with a negative predictive value of 91.67% (Table 1).

Histopathological examination revealed majority of the patients to have moderately differentiated tumours (74.6%), followed by poorly differentiated (19.6%) and well differentiated ones (7.8%). 2 patients (1.9%) had involved margins, whereas 23 patients (22.1%) had close margins. 56 patients had bone involvement out of which 9 had superficial erosion and 47 patients (45.2%) had bone destruction. 36 patients (34.6%) had masticator space involvement (involvement of at least one of masseter, medial pterygoid, lateral pterygoid or temporalis muscle). Table 2 depicts all clinicopathological prognostic factors and unadjusted ‘*p*’ values on log rank test showing their impact of LRFS, DFS and OS.

After the multidisciplinary team discussion, 22 patients (21.1%) were kept on follow up, 62 patients (59.7%) received adjuvant RT whereas 20 patients (19.2%) received adjuvant CTRT.

2-year LRFS was 61.8% (95% confidence interval (CI) 51.0%–70.9%) and 5-year LRFS was 52.5% (95% CI = 40.5%–63.1%) in this study. On adjusting for other prognostic indicators, involved margins after resection (hazard ratio (HR) 28.766, *p* 0.006), metastasis to more than one lymph node (HR = 4.68, *p* = 0.027) and PNI (HR = 3.001, *p* = 0.027) showed statistically significant impact on LRFS (Table 3).

We observed a 2-year DFS of 60% (95% CI = 49.3%–69.2%) and a 5-year DFS of 49.6% (95% CI = 37.9%–60.2%). On adjusting for other prognostic indicators, involved margins after resection (HR = 28.859, *p* = 0.008) and metastasis to more than one lymph node (HR = 4.018, *p* = 0.004) showed statistically significant impact on DFS (Table 3).

OS in this cohort at 2 and 5 years was 68.1% (95% CI = 57.0%–77.0%) and 58.6% (95% CI = 46%–69.2%), respectively. Involved margins after resection (HR = 14.139, *p* = 0.023) and metastasis to more than one lymph node (HR = 3.166, *p* = 0.025) showed statistically significant impact on OS (Table 3).

Proportion of close/ involved margins was higher in patients who had pathological involvement of masticator space (13.2% versus 44.4%, *p* = 0.002) (Table 4).

Patients diagnosed pT4b had a 2-year LRFS, DFS and OS (95% CI) of 49.3% (31.4%–64.8%), 44.5% (27.5%–60.2%) and 55.2% (35%–71.5%), respectively. 5-year survival could not be calculated.

## Discussion

Squamous carcinomas of the oral cavity are staged as T4b when the disease invades the masticator space, pterygoid plates, or skull base and/or encases the internal carotid artery and is referred to as very advanced locoregional disease [8]. Although these tumours are generally considered unresectable, the surgeon can achieve clear margins with his surgery in a subset of T4b tumours depending on the location and surgical technique adopted. While disease encasing the internal carotid artery or invading the skull base are generally considered unresectable, disease confined to the masticator space below the mandibular notch are often considered resectable [6].

Anatomically, the masticator space is located behind the retromolar trigone. It is a pyramidal-shaped space with its base toward the skull base and apex at the lower border of the mandible [10]. There are two described ways tumour spread could occur in the masticator space – one by an upward spread along the neurovascular bundle and the other – through the medial side of the masticator space through the loose areolar tissue, buccal space, then to the lateral wall of the maxilla [11–13].

The decision to proceed with surgery in buccal mucosa malignancy is based on preoperative imaging. CECT scan is the preferred modality for buccal mucosa lesions as CECT is better than magnetic resonance imaging (MRI) in detecting subtle erosions of the cortex of the mandible and provides excellent detection and characterisation of the tumour matrix mineralisation [14, 15]. In our study, CECT scan was performed for all patients, and had a sensitivity of 91.67%, with a lower accuracy (31.73%) in identifying masticator space involvement. These findings contrasted with Huang *et al* [16] who demonstrated CECT to be 25% sensitive and 83.3% specific in identifying pterygoid muscle involvement. MRI had a superior sensitivity (50%) but a lower specificity (75%) when compared to the CECT scan in identifying pterygoid muscle involvement. However, the number of patients in the study was small. The addition of MRI to CECT scan may improve the identification of masticator space involvement. However, further studies are required to draw a meaningful conclusion. Huang *et al* [16] recommended fused fluorodeoxyglucose positron emission tomography (PET) and MRI as a more reliable imaging modality for focal invasion assessment and tumour size delineation in advanced OSCC compared to PET/CT, MRI and CECT.

Although protocols involving induction chemotherapy followed by surgery and/or CTRT have been recommended in a few studies, due to the lack of survival benefit and the extent of surgery after tumour reduction following chemotherapy, surgery followed by adjuvant radiation or CTRT remains the standard of care as curative-intent treatment. For T4b tumours surgery is technically challenging, leading to triaging many of these patients with non-surgical modalities [5, 9, 17–19]. However, recent publications have advocated initial surgery followed by adjuvant treatment for resectable T4b lesions. This is associated with a better 5-year DFS and OS [3]. Our study affirms this with a 5-year LRFS and DFS of 52.5% and 49.6%, respectively.

Mair *et al* [20] reported favourable disease outcomes in T4b buccal mucosa cancer patients treated by multimodality treatment of primary surgery and adjuvant RT or concurrent CTRT. The loco-regional control, DFS and OS were 68.2%, 54.7% and 48.7% respectively, which are comparable favourably with this study. Their cohort excluded patients with supra-notch disease. The study observed significant difference in oncologic outcome with respect to adequacy of surgical margin related to local control (49.6% versus 41.1%, *p* = 0.025) and disease-free survival (65.3% versus 42%, *p* = 0.035); confirming the importance of achieving uninvolved surgical margins when undertaking primary surgery.

Our study, in agreement with Mair *et al* [20] found out that the initial clear surgical margin of resection was favourably affecting LRFS, DFS and OS. In addition, metastasis to two or more cervical lymph nodes was associated with poorer survival outcomes.

**Figure 1.**
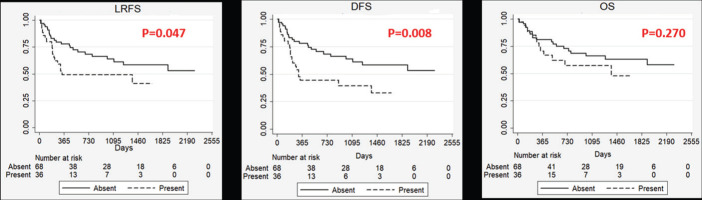
Kaplan–Meier Curves showing impact of masticator space involvement on survival outcomes. LRFS: Locoregional recurrence free survival, DFS: Disease free survival, OS: Overall survival, P: p value on log rank test. Absent: Masticator space not involved of histopathology, Present: Masticator space involved on histopathology (pT4b).

Our study showed a poorer LRFS and DFS in patients with pathological involvement of masticator space (Figure 1). Also, patients with pathological involvement of masticator space showed higher proportion of patients with a close or involved margin in the postoperative specimen. This could be due to the inherent aggressive nature of these tumours or the unfavourable anatomic location deters the surgeon from reaching around the lesion to achieve clear margin in the masticator space.

Retrospective design and the small number of patients are the limitations of this study. The survival outcomes of pT4b patients would have yielded insightful information but were not analysed due to very small number of patients with true masticator space involvement. This highlights the need for a multicentre study to provide further evidence on treatment of patients with masticator space involvement.

## Conclusion

Upfront surgery is a feasible option for patients with buccal mucosa malignancy with masticator space involvement. Survival outcomes are favourable in patients where resection with clear margins is achieved, and the regional disease limited to a single cervical lymph node.

## List of Abbreviations

DFS: Disease free survival
CECT scan: Computed tomographic scan
CI: 95% Confidence interval
CTRT: Chemo-radiation
ENE: Extra nodal extension
HR: Hazard ratio
LRFS: Locoregional recurrence free survival
LVI: Lympho vascular invasion
MRI: Magnetic resonance imaging
OS: Overall survival
OSCC: Oral squamous cell carcinoma
PNI: Perineural Invasion
RT: Radiotherapy

## Conflicts of interest

None.

## Funding

This study did not receive any external funding.

## Figures and Tables

**Table 1.** Efficacy of CECT scan in identifying masticator space involvement.

	Masticator space involved on HPR	Masticator space free on HPR
**Masticator space involved on CECT**	33	68
Masticator space free on CECT	3	0
Total	36	68
Sensitivity	91.67%	
Specificity	------	
Positive predictive value	32.67%	
Negative predictive value	-------	
Accuracy	31.73%	
Likelihood ratio	91.67%	

CECT: Contrast enhanced computed tomography scan; HPR: Final histopathology report.

**Table 2.** Effect of demographic and histopathological parameters on LRFS, DFS and OS. (*N* = 104).

Parameter	*n* (%)	LRFS*	DFS*	OS*
Gender				
Male	81 (77.9)	0.221	0.335	0.107
Female	23 (22.1)			
Masticator space involvement on CECT scan				
Present	101 (97.1)	0.323	0.374	0.384
Absent	3 (2.9)			
Histopathological parameters				
Differentiation				
Well differentiated	8 (7.8)	0.018	0.029	0.012
Moderately differentiated	74 (74.6)			
Poorly differentiated	20 (19.6)			
Margin status				
Free (>5 mm)	79 (76)	<0.001	<0.001	0.002
Close (1–4.9 mm)	23 (22.1)			
Involved (<1 mm)	2 (1.9)			
LVI				
Absent	62 (59.7)	0.044	0.010	0.314
Present	42 (40.3)			
PNI				
Absent	47 (45.2)	<0.001	<0.001	0.005
Present	57 (54.8)			
Bone involvement				
Absent	48 (46.1)	0.328	0.295	0.048
Superficial erosion	9 (8.7)			
Cortical erosion	47 (45.2)			
Masticator space involvement in final histopathology				
Absent	68 (65.4)	0.047	0.008	0.270
Present	36 (34.6)			
ENE				
Absent	92 (88.5)	0.616	0.848	0.569
Present	12 (11.6)			
pT stage				
pT1	7 (6.7)	0.225	0.069	0.286
pT2	21 (20.1)			
pT3	7 (6.7)			
pT4a	33 (31.7)			
pT4b	36 (34.6)			
pN stage				
pN0	47 (45.2)	<0.001	<0.001	0.007
pN1	13 (12.5)			
pN2a	0			
pN2b	38 (36.5)			
pN2c	5 (4.9)			
pN3	1 (1)			
Adjuvant treatment				
Follow up	22 (21.1)	0.601	0.625	0.605
Adjuvant RT	62 (59.7)			
Adjuvant CTRT	20 (19.2)			
Continuous variables				
	Madian (range)	LRFS^#^	DFS^#^	OS^#^
Age (years)	52.5 (27–81)	0.840	0.941	0.873
T size (in cm)	3.5 (0.5–9.5)	0.049	0.100	0.015
Depth of invasion (in cm)	1.6 (0.1–7.2)	0.012	0.004	0.001

* Unadjusted ‘*p*’ value on Log rank test.

# Unadjusted ‘*p*’ value on cox logistic regression.

**Table 3.** Adjusted HR and their significance.

Factor	LRFS		DFS		OS	
	HR (95% CI)	*p* value	HR (95% CI)	*p* value	HR (95% CI)	*p* value
Differentiation						
Well differentiated	1	---	1	---	1	---
Moderately differentiated	0.890 (0.095–8.337)	0.919	0.902 (0.099–8.256)	0.928	0.973 (0.092–10.334)	0.982
Poorly differentiated	2.692 (0.260–27.851)	0.406	2.304 (0.222–23.858)	0.484	3.982 (0.330–48.039)	0.277

Margin status						
Free (>5 mm)	1	---	1	---	1	---
Close (1–4.9 mm)	2.018 (0.850–4.787)	0.111	1.739 (0.762–3.970)	0.189	2.262 (0.933–5.483)	0.071
Involved (<1 mm)	28.766 (2.625–315.271)	0.006	24.859 (2.350–262.881)	0.008	14.139 (1.434–139.362)	0.023
						
LVI	0.603 (0.231–1.567)	0.299	0.988 (0.418–2.332)	0.978	---	---
						
PNI	3.001 (1.135–7.938)	0.027	2.320 (0.945–5.697)	0.066	1.828 (0.721–4.633)	0.204
Bone invasion						
No bone erosion	—	—	—	—	1	—
Superficial erosion	—	—	—	—	1.055 (0.285–3.901)	0.936
Cortical invasion	—	—	—	—	0.759 (0.306–1.882)	0.552

Masticator space invasion on histopathology	0.538 (0.226–1.280)	0.161	0.730 (0.327–1.629)	0.443	—	—

pN stage						
pN0	1	—	1	—	1	—
pN1	1.661 (0.390–7.083)	0.492	1.306 (0.302–5.641)	0.721	0.581 (0.099–3.417)	0.548
pN2b	4.680 (1.721–12.725)	0.027	4.018 (1.543–10.464)	0.004	3.166 (1.152–8.699)	0.025
pN2c	2.675 (0.463–15.452)	0.272	2.381 (0.411–13.772)	0.333	2.479 (0.472–13.030)	0.284
pN3	0.762 (0.031–18.957)	0.869	0.870 (0.036–21.204)	0.932	2.984 (0.162–55.106)	0.462

T size (in cm)	1.292 (0.988–1.691)	0.061	—	—	1.139 (0.887–1.462)	0.308

Depth of invasion (in cm)	1.101 (0.791–1.432)	0.567	1.312 (1.022–1.685)	0.033	1.192 (0.841–1.690)	0.324

**Table 4.** Comparison of margin status in patients with masticator space involvement.

Masticator space involvement on histology	Margin status		
	Free (>5 mm)	Close (1–4.9 mm)	Involved (<1 mm)
Absent (*n* = 68)	59 (86.7%)	8 (11.8%)	1 (1.5%)
Present (*n* = 36)	20 (55.6%)	15 (41.7%)	1 (2.8%)
*p* value	0.002 (on Pearson’s chi square test)		

## References

[1] Mathur P, Sathishkumar K, Chaturvedi M (2020). Cancer Statistics, 2020: Report From National Cancer Registry Programme, India. JCO Glob Oncol.

[2] Pillai V, Yadav V, Kekatpure V (2019). Prognostic determinants of locally advanced buccal mucosa cancer: do we need to relook the current staging criteria?. Oral Oncol.

[3] Liao CT, Wen YW, Lee SR (2017). Clinical outcomes of Taiwanese patients with cT4 oral cavity squamous cell carcinoma: toward the identification of the optimal initial treatment approach for cT4b patients. Ann Surg Oncol.

[4] Handschel J, Naujoks C, Depprich RA (2012). CT-scan is a valuable tool to detect mandibular involvement in oral cancer patients. Oral Oncol.

[5] Pfister DG, Spencer S, Adelstein D (2020). Head and neck cancers, version 2.2020, NCCN clinical practice guidelines in oncology. J Natl Compr Canc Netw.

[6] Liao CT, Ng SH, Chang JT (2007). T4b oral cavity cancer below the mandibular notch is resectable with a favorable outcome. Oral Oncol.

[7] Trivedi NP (2018). Oral cancer involving masticator space (T4b): review of literature and future directions. Head Neck.

[8] Edge SB, Compton CC (2010). The American Joint Committee on Cancer: the 7th edition of the AJCC cancer staging manual and the future of TNM. Ann Surg Oncol.

[9] Bossi P, Lo Vullo S, Guzzo M (2014). Preoperative chemotherapy in advanced resectable OCSCC: long-term results of a randomized phase III trial. Ann Oncol.

[10] Fernandes T, Lobo JC, Castro R (2013). Anatomy and pathology of the masticator space. Insights Imaging.

[11] Kowalski LP, Hashimoto I, Magrin J (1993). End results of 114 extended "commando" operations for retromolar trigone carcinoma. Am J Surg.

[12] Antoniades K, Lazaridis N, Vahtsevanos K (2003). Treatment of squamous cell carcinoma of the anterior faucial pillar-retromolar trigone. Oral Oncol.

[13] Pinsolle J, Demeaux H, Coustal B (1992). Results of surgical treatment of T3 and T4 tumors of the oral cavity and oropharynx. Am J Surg.

[14] Galli F, Flor N, Villa C (2010). The masticator space. Value of computed tomography and magnetic resonance imaging in localisation and characterisation of lesions. Acta Otorhinolaryngol Ital.

[15] Som PM, Curtin HD, Som PM, Curtin HD (2003). Parapharyngeal and masticator space lesions. Head and neck imaging.

[16] Huang SH, Chien CY, Lin WC (2011). A comparative study of fused FDG PET/MRI, PET/CT, MRI, and CT imaging for assessing surrounding tissue invasion of advanced buccal squamous cell carcinoma. Clin Nucl Med.

[17] Liao CT, Wang HM, Ng SH (2006). Good tumor control and survivals of squamous cell carcinoma of buccal mucosa treated with radical surgery with or without neck dissection in Taiwan. Oral Oncol.

[18] Zhong LP, Zhang CP, Ren GX (2013). Randomized phase III trial of induction chemotherapy with docetaxel, cisplatin, and fluorouracil followed by surgery versus up-front surgery in locally advanced resectable oral squamous cell carcinoma. J Clin Oncol.

[19] Joshi A, Patil VM, Noronha V (2013). Is there a role of induction chemotherapy followed by resection in T4b oral cavity cancers?. Indian J Cancer.

[20] Mair MD, Sawarkar N, Nikam S (2018). Impact of radical treatments on survival in locally advanced T4a and T4b buccal mucosa cancers: Selected surgically treated T4b cancers have similar control rates as T4a. Oral Oncol.

